# Filling gaps in type 1 diabetes and exercise research: a scoping review and priority-setting project

**DOI:** 10.1136/bmjdrc-2019-001023

**Published:** 2020-03-04

**Authors:** Nika M D Klaprat, Nicole Askin, Andrea MacIntosh, Nicole Brunton, Jacqueline L Hay, Jane E Yardley, Seth D Marks, Kathryn M Sibley, Todd A Duhamel, Jonathan M McGavock

**Affiliations:** 1 Diabetes Research Envisioned and Accomplished in Manitoba (DREAM) Theme, University of Manitoba, Children’s Hospital Research Institute of Manitoba, Winnipeg, Manitoba, Canada; 2 Rady Faculty of Health Sciences, University of Manitoba, Winnipeg, Manitoba, Canada; 3 Neil John Maclean Library, University of Manitoba, Winnipeg, Manitoba, Canada; 4 Albrechtsen Research Centre, St Boniface Hospital Research, Winnipeg, Manitoba, Canada; 5 Faculty of Kinesiology, Sport and Recreation, University of Alberta—Augustana Campus, Camrose, Alberta, Canada; 6 Alberta Diabetes Institute, Edmonton, Alberta, Canada; 7 Diabetes Education Resource for Children and Adolescents, Winnipeg, Manitoba, Canada; 8 George and Fay Yee Centre for Healthcare Innovation, Winnipeg, Manitoba, Canada; 9 Faculty of Kinesiology and Recreation Management, University of Manitoba, Winnipeg, Manitoba, Canada; 10 Diabetes Action Canada SPOR Network, Toronto, Ontario, Canada

**Keywords:** patient-oriented research, type 1, exercise

## Abstract

Our team examined the characteristics of patient engagement (PE) practices in exercise-based randomized trials in type 1 diabetes (T1D), and facilitated T1D stakeholders in determining the top 10 list of priorities for exercise research. Two methodological approaches were employed: a scoping review and a modified James Lind Alliance priority-setting partnership. Published (Medline, Embase, CINAHL and Central databases) and grey literature (www.clinicaltrials.gov) were searched to identify randomized controlled trials of exercise interventions lasting minimum 4 weeks and available in English. We extracted information on PE and patient-reported outcomes (PROs) to identify if patient perspectives had been implemented. Based on results, we set out to determine exercise research priorities as a first step towards a patient-engaged research agenda. An online survey was distributed across Canada to collect research questions from patients, caregivers and healthcare providers. We qualitatively analyzed submitted questions and compiled a long list that a 12-person stakeholder steering committee used to identify the top 10 priority research questions. Of 9962 identified sources, 19 published trials and 4 trial registrations fulfilled inclusion criteria. No evidence of PE existed in any included study. Most commonly measured PROs were frequency of hypoglycemia (n=7) and quality of life (n=4). The priority-setting survey yielded 194 submitted research questions. Steering committee rankings identified 10 priorities focused on lifestyle factors and exercise modifications to maintain short-term glycemic control. Recent exercise-based randomized trials in T1D have not included PE and PROs. Patient priorities for exercise research have yet to be addressed with adequately designed clinical trials.

## Introduction

Exercise provides numerous health benefits for individuals with type 1 diabetes (T1D)^1^ and is an important component of diabetes self-management.^2^ Despite vast health benefits, only one-third of people with T1D meet minimum recommendations for regular exercise to achieve health benefits.^3^ The unique barriers to exercise for people with T1D^4 5^ are severe, particularly loss of glycemic control and hypoglycemia. With few evidence-based strategies available to overcome these barriers, novel approaches are needed to improve the efficacy of future exercise trials to address patient-relevant concerns.

Including patients in designing and delivering research studies can help address patient-relevant gaps in clinical research^6 7^ such as understanding barriers to uptake of exercise among people with T1D. Patient-oriented research, being ‘a continuum of research that engages patients as partners, focuses on patient-identified priorities and improves patient outcomes’,^8^ is becoming a priority for clinical trial funding and design, but has had little traction in exercise and T1D science.^9^ Patients with T1D have previously been involved in a range of patient engagement (PE)^10–12^ or priority-setting activities^13^ to optimize blood glucose self-management and overall health. Notably, these studies have not centred on exercise research. The current status of PE in setting priorities for and conducting research within exercise science for patients with T1D remains relatively unknown.

This study addresses these gaps in the literature. First, we conducted a scoping review of exercise training randomized trials for patients with T1D to map patient engagement within recent trials. Informed by these results, we then engaged patients with T1D, caregivers and healthcare providers in conducting a modified James Lind Alliance (JLA) model^14^ of research prioritization to identify the most important questions about exercise and health.

## Study designs and research methods

### Study 1: Scoping review of physical activity/exercise randomized trials and type 1 diabetes

We conducted a scoping review of published and grey literature available from the past 20 years to determine in a single narrative analysis: (1) the characteristics of exercise training interventions delivered to people with T1D and (2) the extent patient partners or patient-reported outcomes (PROs) were involved in study development. The primary question guiding this review was ‘Is there evidence of patient perspectives being incorporated in developing or implementing long-term exercise training trials for individuals with T1D?’

This review was conducted following the five-stage Arksey and O’Malley framework^15^ and formatted in accordance with Preferred Reporting Items for Systematic Reviews and Meta-Analyses Scoping Review reporting guidelines.^16^ A review protocol was not published prior to its conduct. Our team operationalized PE as per the Canadian Institutes of Health Research definition as being ‘meaningful and active collaboration in governance, priority-setting, conducting research and knowledge translation’.^8^ In contrast, PROs are defined as ‘a measurement based on a report that comes directly from the patient (ie, study subject) about the status of a patient’s health condition without amendment or interpretation of the patient’s response by a clinician or anyone else’.^17^ Therefore, measurement of PROs does not necessarily directly involve engaging patients in the research process, but these outcomes typically reflect those preferred by the patient population.^18^

#### Data sources

Information for this review was collected from published and grey literature. A trained university librarian (NA) developed and implemented search strategies (online supplementary 1). The published literature search strategy was developed for Medline and adapted to Embase, CINAHL and Central databases, respectively. Database searches were conducted on 22 August 2018, updated on 16 May 2019, and restricted to articles published in the preceding 20 years (January 1998 to May 2019, inclusive). Citations and abstracts for identified publications were uploaded to Rayyan QCRI review management software^19^ for screening.

10.1136/bmjdrc-2019-001023.supp1 Supplementary data

Additionally, the Clinical Trials online registry (www.clinicaltrials.gov) was searched to identify ongoing trials (grey literature). Included trial registrations satisfied the same inclusion criteria as published literature according to information provided. Registrations with related publications were added to the published literature analysis; otherwise, detailed aims and protocols were recorded.

#### Inclusion/Exclusion screening

We included randomized controlled trials of exercise training for individuals with T1D, limited to interventions lasting 4 weeks or longer. We excluded short-term trials and laboratory-based acute exercise studies, which are common in exercise science as they are largely mechanistic studies with few patients, focused on physiological responses to exercise and not long-term adaptations to exercise. Interventions providing education to support behavior change without directly implementing an exercise program were also excluded. Full-text sources had to be available in the English language. These inclusion/exclusion criteria were applied because our purpose was to assess the influence of patient perspectives within high-quality, controlled studies which may inform exercise guidelines or clinical recommendations ultimately provided to patients.

Screening of published literature occurred in duplicate. Two reviewers independently screened titles and abstracts from the initial (NMDK and AM) and updated searches (NMDK and NB). Conflicts following independent screening were resolved through discussions between reviewers. Full-text versions of potentially eligible articles were searched and uploaded to Rayyan software. Full-text screening was undertaken by both reviewers concurrently. The principal investigator (JMMG) was consulted throughout screening where disagreement remained after reviewer discussions.

#### Data extraction

Publications were randomly divided between two coauthors to independently extract data (NMDK and AM). Where further information was required,^20 21^ corresponding authors were contacted electronically. A data extraction form was developed for all published and grey literature data to identify: publication information, participant characteristics, intervention details (frequency, intensity, type, time and intervention duration) and measured outcomes (extracted as per reporting within each study). Reviewers noted evidence of patient engagement if authors declared involvement of people with T1D in research question selection, study design, recruitment, data collection, data analysis and interpretation or manuscript preparation. Finally, reviewers recorded whether PROs were measured, including quality of life, diabetes distress, perceived competence, problem areas, self-management behaviors, frequency of glycemic symptoms and several core outcomes identified by the Irish D1 Now Study.^12^ These PROs were collected as a proxy for reflecting patient-relevant research interests, which the Cochrane Collaboration discussed as an appropriate approach since PROs reflect patient health-related experiences.^18^ Measures included in the D1 Now core outcome set were considered PROs throughout this review, as patients and other stakeholders were involved in their selection.^12^

### Study 2: Priority-setting partnership for research in type 1 diabetes and exercise

Following the scoping review, our team conducted a priority-setting partnership with patients, caregivers and healthcare providers living or working with T1D. We adapted the JLA model of priority-setting, which is supported by the Cochrane Collaboration.^22^ This study is reported in accordance with GRIPP2 reporting standards for patient and public involvement in research.^23^

The JLA approach to priority-setting is a multistage, mixed-methods research design,^14 24^ which we modified to identify priorities for exercise and T1D. The role of the first author was similar to that of a JLA independent advisor.

#### Phase I

We created an online survey using REDCap Surveys server hosted at the University of Manitoba^25 26^ to collect responses to the item, “What questions about physical activity and T1D would you like to see answered by research?” Four open-text response boxes were available for respondent submissions. Demographic (age, province of residence and relationship to diabetes) and related patient information (current age, age of diagnosis, gender and ethnic identity) were also collected. The survey was distributed across Canada for 6 months. Survey respondents were recruited through communications from partnered diabetes advocacy organizations (JDRF, Diabetes Canada and Diabetes Action Canada), a paid social media advertising campaign and posters in diabetes clinics or wellness centers in several urban centers throughout Canada. Concurrently, 12 individuals (8 patients, 3 caregivers and 4 healthcare providers) were recruited to participate in a steering committee. Steering committee members were recruited via maximum variation sampling methods^27^ from across Manitoba to represent variance across age, sex, ethnicity and rural versus urban residence. Potential members were identified through posters, local chapters of diabetes advocacy organizations and lab contact information provided on submission of the online survey. Typically, a JLA steering committee will include members from advocacy organizations or charities supporting the appropriate health condition. Our goal was to emphasize the control of those directly affected by T1D in setting research priorities; therefore, our steering committee did not include representatives from these organizations. This steering committee was responsible for prioritizing submitted questions.

#### Phase II

On survey closure (February 2019), demographic information was extracted directly from the REDCap database. Submitted questions were uploaded to NVivo V.12 analysis software. A graduate student trained in qualitative research methods (NMDK) analyzed submissions by conventional content analysis methodology.^28^ This qualitative methodology can be used when there is limited knowledge supporting a given topic, as is the case in patient-oriented research questions about T1D and exercise. Data analysis is inductive and uninformed by preconceptions of possible themes or categories. Conventional content methodology was primarily used in this project in creating themes of submitted questions to facilitate filtering out repeated questions and create a concise long list of submitted research questions. Four senior investigators (JMMG, TAD, SDM and KMS) were consulted throughout analysis to review results and provide guidance for complicated decisions. A long list of 38 research questions was developed following phase II, in line with JLA recommendations.^14^ An additional literature search was conducted to identify if any of the long-listed questions had been addressed by a systematic review or meta-analysis in the past 3 or 10 years (noted separately). The JLA traditionally removes questions that have been recently addressed by a high-level synthesis. However, our team noted which questions had synthesized data available but maintained these questions within the long list, to distinguish between lack of available data and knowledge translation deficiencies.

#### Phase III

Long-listed questions were distributed to the steering committee in a randomized order. Each committee member reviewed the list and ranked their top 10 questions in order of 1 (most important) to 10 (10th most important). Rankings were returned after the 2-week review period by email in word documents encrypted with personalized passwords for each member. Rankings were collated through an inverted points-based system whereby top-ranked questions of each member were denoted 10 points, and each successive ranking received one less point. Total points for each question were summed and each question receiving 10 or more points (in keeping with the JLA T1D partnership) was short-listed for further prioritization. The process followed in this project slightly contrasts that of the JLA, where the long list is distributed to the general public rather than the steering committee. This modification was made for two reasons: 1) our team was unable to recontact participants from the initial anonymous survey to rank the synthesized questions and 2) due to the modification of the steering committee composition, our team wanted to facilitate deeper, meaningful involvement of patients, caregivers and healthcare providers on our committee.

#### Phase IV

A 1 day in-person workshop for steering committee members was facilitated by the research team (NMDK, JMMG, NB and JLH) to create the final top 10 list of research priorities in exercise and T1D. The goal of the workshop was to reach consensus on priority research questions, defined as every member having at least 80% agreement with the resulting top 10 list. The workshop began with an independent prioritization activity, where committee members were asked to individually select, in no particular order, their top and bottom three questions from the short list.

Steering committee members were divided into three smaller groups, where each group ranked all short-listed questions in order of importance using printed cards. Anonymous notes were provided to each small group, reflecting the results of their independent selections. Small group exercises were repeated three times with different group members, and the research team collated rankings from all groups after each round and presented results to the full committee. After rounds two and three, each member anonymously rated their level of agreement from 1 to 10 to determine the level of consensus across all committee members.

### Patient and public involvement

As per the IAP2 Spectrum of Public Participation,^29^ patients and public stakeholders were consulted to prioritize research questions throughout three of four priority-setting project phases. All research question data were developed and prioritized by patients, caregivers and healthcare providers of individuals with T1D. These research questions will inform our future research agenda, for which we plan to collaborate with stakeholder partners.

## Results

### Study 1: Scoping review

**Figure 1:**
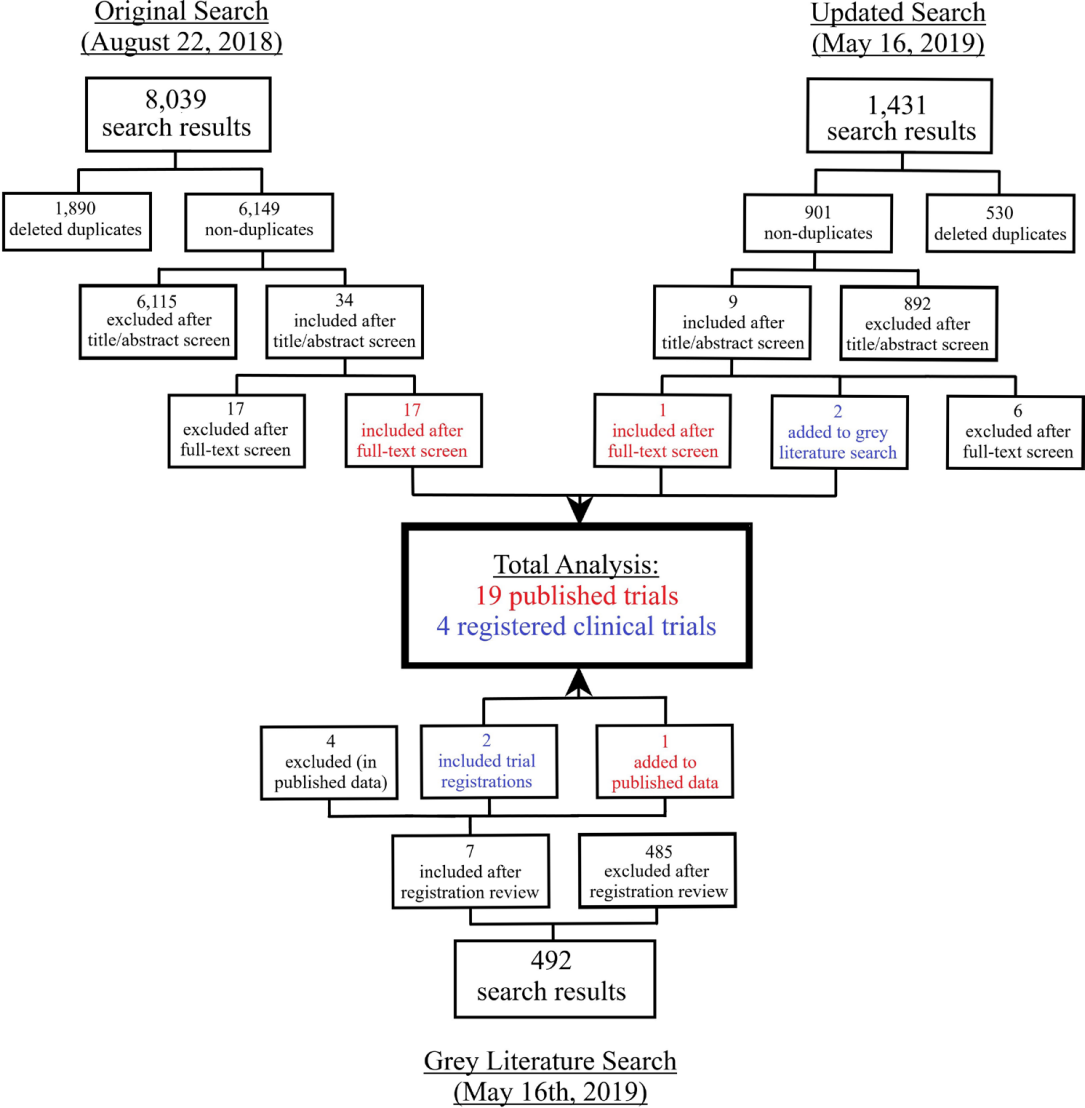
Study flow diagram.

The published literature search yielded 9470 citations (figure 1). Following independent deletion of duplicates and title and abstract screening, 43 citations remained for full-text review. Twenty citations remained after full-text review, 2 of which were clinical trial registrations and added to the grey literature, leaving 18 published articles. Grey literature searches identified 492 possibly relevant registrations. After eligibility screening, seven fulfilled inclusion criteria; however, four were excluded as relevant articles were already included in the published literature analysis. One registration provided a full-text publication, and was thus added to the published literature. Therefore after screening, 19 published articles and 4 registered trials were included for analysis.

#### Published literature

Data were available for 890 individuals living with T1D (n=18 trials reporting sample sizes). Among studies providing demographic information, 53% of participants were female (n=17 trials), 61% were youth under the age of 18 years (n=16 trials) with a mean hemoglobin A1c of 8.43% (95% CI: 7.26% to 9.61%, n=13 trials). Most participants were categorized as sedentary or unconditioned at enrollment by the relevant authors (n=12 trials) and had lived with T1D for a mean of 5.5 years (median: 5.4; range: 2.9–24.4 years, n=14 studies).

Intervention summaries are provided in online supplementary 2. The majority of trials compared aerobic or combined aerobic and resistance training to a non-exercise control group. Interventions were delivered under supervised conditions by a kinesiologist or equivalent, for a median of 60 min/session (range: 10–90 min), three times/week (range: 1–5 times) for 20 weeks (range: 6 weeks–4 years). Twenty-three outcomes were reported across the 19 trials (online supplementary 3), of which nearly all focused on physiological factors, with glucose control and predictors of cardiovascular health being most common.

10.1136/bmjdrc-2019-001023.supp2 Supplementary data

10.1136/bmjdrc-2019-001023.supp3 Supplementary data

There was no evidence that any trials conducted to date engaged individuals with lived experience of T1D. Number of hypoglycemic events was the most commonly discussed PRO (n=7 studies).^20 21 30–34^ Additionally, episodes of diabetic ketoacidosis were indirectly observed in two studies^32 34^ (number of adverse events) and quality of life was measured in three studies but only reported in two^31 35 36^ (specified different scales: validated French version of Diabetes Quality of Life Scale and the Dutch version of General Health Survey Short Form-36).

#### Clinical trials registrations

Across the four identified registered clinical trials^37–40^ (online supplementary 4), there are plans to collect data from 187 participants. Three trials are exclusively enrolling youth participants (under 18 years of age). Planned exercise sessions frequency is a median of 3 times/week (range: 2 times/week–3 times/day, n=3 trials) for 45 min/session (range: 3–50 min, n=3 trials) lasting 15.1 weeks (range: 12–32 weeks, n=4 trials). One trial^39^ has reached target enrollment and anticipates publishing by the end of 2019. No trial registration described partnerships with stakeholders in developing or implementing the study. Quality of life is the only PRO explicitly disclosed in one trial^40^ (scale is not specified).

10.1136/bmjdrc-2019-001023.supp4 Supplementary data

### Study 2: Priority-setting exercise

The online survey was available and advertised to the public between July 2018 and January 2019. We collected responses from 115 individuals across nine Canadian provinces. Respondents were a mean age of 40.9 (±15.1) years, and the majority (73.9%) identified as a patient with T1D. The remainder identified as caregivers (15.7%), friends (7.0%) or healthcare providers (12.2%), with some respondents identifying as more than one category (8.7%). More females completed the survey (63.4%) than males, and no participants identified as a non-binary gender. Most patients identified as having Canadian ethnic origins (74%), with the next largest samples having European Canadian (15%), Métis (4%) and Caribbean Canadian (3%) roots. Participants selected the category that best fit their ethnic self-identity, with the ‘Canadian’ category reflecting individuals who did not identify with historical familial roots from other nationalities.

**Figure 2:**
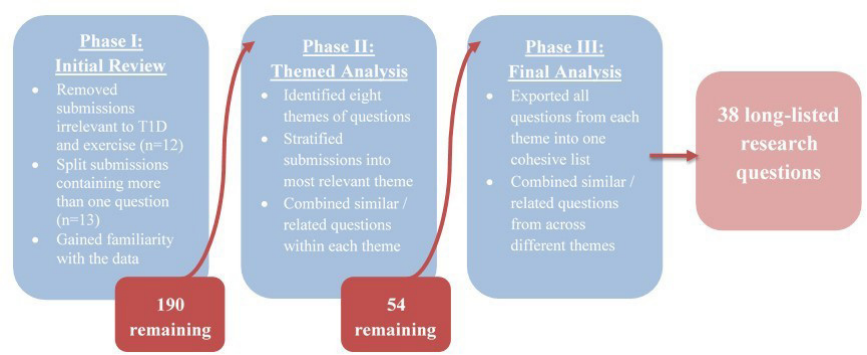
Qualitative analysis process.

Of the 115 respondents, 100 submitted at least one research question, producing a total of 194 submissions. After qualitative analysis (figure 2), 38 research questions were included in the phase III long list and distributed to steering committee members for review. We received 100% of our steering committee rankings between 21 February and 8 March 2019. Twenty-four questions were short-listed after receiving 10 or more points in collated rankings.

Eleven of 12 steering committee members attended the phase IV workshop on 6 April 2019. The workshop lasted approximately 6 hours, throughout which three rounds of small group discussions occurred. Consensus on an aggregated top 10 list was not achieved following the small group sessions. Therefore, we conducted a postworkshop analysis using third round rankings from small groups as votes to supplement the list from the second round. Prioritized questions were removed, added or shuffled based on the majority of votes from third round small group discussions. This analysis resulted in the final top 10 list of research priorities for T1D and exercise (table 1).

**Table 1:** Research priorities for exercise and type 1 diabetes

Position #	Research question
1	What explains the variation in responses that the same person can experience doing the same exercise between different days?
2	Which is the best for maintaining glycemic stability and glucose tolerance: aerobic training, strength training or a combination of both? If a combination, does the order matter?
3	What modes of exercise (ie, activity types, such as walking, cycling, weightlifting, rock climbing, etc) produce the best health benefits while maintaining tight glycemic control?
4	What dietary plans can safely and effectively be followed for an active lifestyle in type 1 diabetes without compromising pre-exerise and postexercise glycemic control?
5	What is the optimal time of day and exercise prescription (example: how often, what type, how intense) in order to maintain ideal glycemic control and insulin sensitivity?
6	What is the best method of preventing postexercise hypoglycemia or hyperglycemia?
7	Will certain glycemic ranges before starting exercise consistently result in hypoglycemia or hyperglycemia?
8	What effect can various levels of hydration have on blood sugar levels during and after exercise?
9	How does hypoglycemia or hyperglycemia affect muscle growth and strength training progress, or vice versa?
10	What is the effect of climate/temperature on blood sugar control during exercise and what causes this effect?

## Discussion

This study examined the characteristics of randomized trials and patient priorities for exercise science research for people with T1D. In our scoping review, we determined that patient engagement methods and PROs have not been historically used to inform exercise-based interventions. Guided by these results, we facilitated a priority-setting project with T1D stakeholders to identify priority research questions pertaining to exercise and health. We identified that patients and caregivers are interested in modalities and strategies to exercise safely and maintain glucose control. Collectively, these findings provide a novel patient-centered rationale for designing future randomized trials of exercise interventions for people with T1D.

### Previous literature

This is the first scoping review of exercise randomized trials for individuals with T1D designed to determine if patient engagement exists in exercise and T1D literature. This topic was not addressed in recent systematic reviews of exercise training and health outcomes in people with T1D.^41–43^ We found that exercise randomized trials published or being delivered for individuals with T1D did not focus on stakeholder engagement. This gap is not exclusive to trials of T1D and exercise. A scoping review of priority-setting practices in all health research found only 27 studies engaged patients in identifying research topics, and 12 in identifying specific research questions.^44^ Many studies simply inferred stakeholder priorities from qualitative data. Additionally, most trials engaging stakeholders do not integrate multiple stakeholders’ perspectives (ie, patients, clinicians, caregivers, etc) in the prioritization process. This is an important consideration when engaging stakeholders in research, as stakeholders with different experiences of a health condition may have different priorities for research topics or outcomes.^45^ Engaging T1D stakeholders is a significant gap in exercise science literature and should be considered within future randomized trials.

As of yet, PRO reporting has been minimal across T1D and exercise randomized controlled trials. This may be due to the lack of agreement on a core list of PROs to be measured in this field. Unfortunately, the core list of PROs determined by the D1 Now study in Ireland^12^ includes outcomes that are not relevant to exercise interventions (eg, level of clinic engagement). It may be prudent for researchers and stakeholders to co-produce a core list of PROs for T1D and exercise in the near future. Some exercise trials have measured PROs as primary or secondary outcomes,^31 35 46^ a practice we hope to see translated into more randomized controlled trial designs as well. However, it is important to recognize that PRO measurement may not be suitable for all exercise trials, particularly those with small sample sizes, and qualitative interviews investigating intervention acceptability may be more appropriate.

The stakeholders engaged as steering committee members were recruited to reflect variance across age, sex, ethnicity and rural versus urban residence categories. These four variables were identified from previous studies that used these sampling methods^47–49^ and were selected by consensus from five members of the research team (NMDK, SDM, KMS, TAD and JMMG). It is important to recognize that other social determinants outside of these selected variables could have impacted the resulting list of prioritized questions (ie, household income, religion, duration of diabetes, etc). However, in keeping with recommendations from the JLA regarding steering committee size, only a select number of factors could be used in recruitment to adequately reflect differences within each. Despite the inability to recruit steering committee members across additional social determinants, our survey sample spanning nine provinces in Canada provides some confidence in the representativeness of the resulting priorities.

As with any research involving group-based activities, it was important for our team to consider the possible effects of perceived power dynamics in small group discussions and ranking exercises. This was particularly important since youth and multiple stakeholder types (both patients and healthcare providers) were involved in this project. As per the JLA guidebook,^14^ an adapted nominal group technique^50^ guided the final workshop procedures. This permitted anonymous voting on individual’s level of agreement with aggregated lists in between each small group discussion session, to ameliorate the risk of individuals simply agreeing with decisions made by fellow group members. Additionally, a research team member was assigned to each small group (NB, JLH, JMMG) during discussions as a moderator to support fair engagement among all group members. The research coordinator (NMDK) floated between all groups to help facilitate discussions and affirm rankings were reflective of stakeholder discussions.

Patient engagement and priority-setting projects identifying important research topics from stakeholders’ perspectives are becoming more common within clinical research.^44 51 52^ This project revealed that stakeholders are largely concerned with short-term outcomes, strategies to prevent hypoglycemia and stabilizing short-term glucose control. This contrasts the JLA T1D treatments project,^13^ where prioritized questions focused on long-term outcomes including adverse effects of various insulin analogues or potential cognitive impacts of living with T1D. This difference may indicate some uncertainty felt by stakeholders regarding the safety of exercise given their individual situation. Fear of short-term health complications is a common barrier to regular exercise among people with T1D.^5^ This fear itself has a range of health implications including reduced physical activity,^53^ increased glycemic variability,^53^ poorer sleep patterns^54^ and reduced quality of life.^54^ Although guidelines and consensus statements about prevention of postexercise hypoglycemia exist,^55 56^ the literature on which these recommendations are based has limitations. As this is the first investigation into patient priorities in T1D and exercise, previous research may not have been intentionally directed towards established patient-identified questions. Future randomized trials should focus on stakeholder priorities to provide optimally relevant recommendations to individuals with T1D.

### Strengths and weaknesses

This study was strengthened by using two complimentary methods to identify and address the gap of patient priority-setting and engagement in exercise science for individuals with T1D. The scoping review approach supported a rigorous and systematic search of published and unpublished literature sources. This search strategy provided the richest base possible to analyze the narrative of previously conducted studies using a patient-oriented research lens. The narrative approach of scoping reviews enabled discussion of the existing literature beyond summaries and quantitative meta-analyses that have recently been conducted by other authors in the field.^42 57^ The priority-setting project followed a modified JLA approach to priority-setting, and the recognition and support for this model^22^ is a strength for our study. The steering committee recruited for this project were highly engaged (100% phase III participation rate), which served as assurance that a patient-oriented approach to research moving forward would be valued by this stakeholder population. Despite these strengths, several limitations should be addressed. Although scoping reviews do not require a critical analysis of included studies,^16^ this is not a limitation of this review as our purpose was simply to identify instances where stakeholders may have been involved in research decision-making. However, the scoping review was limited to trials published in the English language over the past two decades, therefore some trials engaging patients or other stakeholders may have been missed. Additionally, the authors recognize that reporting of patient engagement is only recently growing within the literature, although still not captured in the Consolidated Standards of Reporting Trials guidelines for reporting randomized controlled trials. As a result, patient engagement practices may have occurred but not described in every publication, particularly within journals having a lower word limit. The EXTOD (EXercise for Type One Diabetes) study serves as one example of various publications addressed different aspects of the research design and outcomes, including qualitative patient engagement practices in the early design phase.^58 59^ However, data for this review were extracted strictly as reported within articles. First authors were not contacted and related publications were not searched for to confirm whether patient engagement played a role within the studies. Future reviews with a similar purpose may want to explore possibilities for this additional data collection. In terms of the priority-setting project, one limitation is that consensus was not achieved in person at the final workshop. Although the JLA Guidebook^14^ mentions that it is not uncommon for consensus to be difficult to achieve and a majority vote can be obtained in these situations, this process had to be conducted in a postworkshop analysis since several members had other commitments.

### Impact of patient engagement

Consulting end users as participants was integral to this study. The developed list of research questions was based on submissions collected directly from and prioritized by end users of research. Our team was able to facilitate meaningful discussions and share perspectives between researchers, patients, caregivers and healthcare providers, without which a very different set of prioritized questions would likely have resulted. The connections formed between our research team and stakeholder partners in this first stage has provided a basis of trust and inclusiveness between the research community and those with lived experience. We will continue to foster these relationships when moving forward in designing clinical trials alongside patient partners that address these research priorities.

## Conclusion

We have outlined the current status of patient engagement in exercise research for individuals with T1D and engaged stakeholders in developing a list of priorities in T1D and exercise research. This list of priorities will be used to guide our future research agenda, and we recognize the need to continue working with stakeholders in designing future research. It will also be critical to re-evaluate priorities as new information becomes available.
