# Supplementary material for: Filling gaps in type 1 diabetes and exercise research: a scoping review and priority-setting project
Source: BMJ Open Diabetes Res Care. 2020 Mar 4;8(1):e001023. doi: 10.1136/bmjdrc-2019-001023 (PMC7059416; doi:10.1136/bmjdrc-2019-001023)
Supplement: Supplementary data [file bmjdrc-2019-001023supp001.pdf]

Scoping Review Search Strategy

MEDLINE (Published Literature) Search Strategy:

exp Diabetes Mellitus, Type 1/
(autoimmune OR brittle OR juvenile OR juvenile-onset OR ketosis prone OR sudden onset OR type 1 OR type I OR insulin dependent) adj3 diabet*.ti,ab,kf
IDDM.ti,ab,kf
T1DM.ti,ab,kf
or/1-4
exp Exercise Therapy/
exp Exercise/
exp Exercise Movement Techniques/
exp "Physical education and training"/
exp Physical fitness/
exp Sports/
exp Running/ or walking/ or swimming/
Yoga/ or Tai Ji/
exp Health Promotion/
exercis*.ti,ab,kf
physical* adj (activ* or conditioning).ti,ab,kf
personal train*.ti,ab,kf
fitness.ti,ab,kf
kinesiotherap*.ti,ab,kf
strength adj3 train*.ti,ab,kf
resistance adj3 train* .ti,ab,kf
weight adj3 (lift* or train*).ti,ab,kf
(run* or walk* or jog* or tai chi or tai ji or yoga or swim* or sport* or aerobic*).ti,ab,kf
((sedentary or lifestyle or life style) adj3 (promot* or counsel* or advis* or educat* or program* or interven* or train* or therap* or prescri* or refer* or motivat* or coach* or encourag*)).ti,ab,kf
or/6-24
randomized controlled trial.pt
controlled clinical trial.pt
(randomized or randomised).ab
placebo.ab
randomly.ab
trial.ab
groups.ab
or/26-32
exp animals/ not humans.sh
33 not 34
5 and 25 and 35
limit 36 to yr="1998 -Current"

CLINICALTRIALS.GOV Search Strategy:

(exercise OR fitness OR physical)
Interventional Studies
Type 1 Diabetes
