# Supplementary material for: Filling gaps in type 1 diabetes and exercise research: a scoping review and priority-setting project
Source: BMJ Open Diabetes Res Care. 2020 Mar 4;8(1):e001023. doi: 10.1136/bmjdrc-2019-001023 (PMC7059416; doi:10.1136/bmjdrc-2019-001023)
Supplement: Supplementary data [file bmjdrc-2019-001023supp002.pdf]

SUPPLEMENTARY 2: INTERVENTION DETAILS OF INCLUDED EXERCISE-BASED CLINICAL TRIALS

**Publication Information**			**Intervention Details**					
Authors	*Journal*	*Year*	*Sample Size*	*Type*	*Frequency*	*Intensity*	*Time/Session*	*Duration*
Aouadi et al[19]	Journal of Sports Medicine and Physical Fitness	2011	33	Aerobic vs control	Arm 1: 2x/week<br>Arm 2: 4x/week	50-65% maximum heart rate	40-50 minutes	6 months
Balducci et al[56]	Journal of Diabetes and Its Complications	2006	21	Aerobic vs control	4x/week	50-85% heart rate reserve	1 hour	4 years
Brazeau et al[18]	Applied Physiology, Nutrition and Metabolism	2014	48	Combined (aerobic, resistance, flexibility) vs control	1x/week	Not reported	60 minutes	3 months
Boff et al[29]	Frontiers in Physiology	2019	27	High intensity interval training vs moderate continuous exercise vs non-exercise control	3x/week	Arm 1: 1 minute @ 80% maximum capacity, 4 minutes @ 50% maximum capacity<br>Arm 2: 50-65% maximum capacity	30 minutes	8 weeks

D'hooge et al[30]	Clinical Rehabilitation	2011	16	Aerobic and resistance vs control	2x/week	60-75% heart rate reserve for aerobic exercise, 12-20 repetition maximum for resistance exercises	70 minutes	20 weeks
Gusso et al[57]	Diabetes Care	2017	50	Mixed aerobic and resistance training vs control	4x/week	Progressed to and sustained 85% maximum heart rate	60 minutes	20 weeks
Heyman et al[34]	Pediatric Exercise Science	2007	16	Combined aerobic and strength vs control	2x/week (one supervised, one unsupervised)	80-90% heart rate reserve	Supervised sessions: 2 hours<br><br>Unsupervised sessions: 1 hour	6 months
Laaksonen et al[58]	Medicine & Science in Sport & Exercise	2000	56	Aerobic exercise	4-5x/week	Start at 50-60% $VO2_{peak}$, progress to 60-80% $VO2_{peak}$	Start at 20-30 minutes, progress to 30-60 minutes	12-16 weeks

Maggio et al[59]	Medicine & Science in Sports & Exercise	2012	27	Mixed aerobic and resistance training vs control	2x/week	Intensity maintained at 140 per minute during jumping or game-based activities	90 minutes	9 months
Mohammed et al[60]	International Journal of PharmTech Research	2016	50	Aerobic exercise	3x/week	60-75% maximum heart rate	40 minutes	3 months
Quirk et al[31]	BMC Pediatrics	2018	13	Group-based circuit activities and DVD dance routine vs non-exercise control	1 or more sessions per week (self-selected)	Not reported	Variable (self-selected sessions)	6 weeks
Roberts et al[61]	Journal of Pediatric Endocrinology and Metabolism	2002	Not reported	Mixed aerobic and anaerobic training vs control	3x/week	160 bpm heart rate for minimum of 30 minutes of session	45 minutes	Total 24 weeks:<br>12 weeks supervised,<br>12 weeks unsupervised

Salem et al[32]	Diabetology & Metabolic Syndrome	2010	196	Mixed exercise program vs control	Arm 1: 1x/week<br><br>Arm 2: 3x/week	Aerobic: 65-85% maximum heart rate<br><br>Anaerobic: 85-95% maximum heart rate	Whole session time not reported (estimate = 70 minutes)	6 months
Sigal et al[62]	Diabetes	2011	66	Aerobic vs Resistance vs Aerobic + Resistance vs Control	3x/week	Aerobic: 75% maximum heart rate Resistance: 8 repetition maximum	Arm 1: 45 minutes<br><br>Arm 2: 3 sets of 8 exercises (estimate = 45 minutes)<br><br>Arm 3: 45 minutes + 3 sets of 8 exercises (estimate = 90 minutes)	6 months
Sigal et al[63]	Abstracts, Canadian Journal of Diabetes	2012	131	Resistance vs control	3x/week	8 repetition maximum	3 sets of 8 exercises	22 weeks
Talakoub et al[64]	Iranian Journal of Nursing and Midwifery Research	2012	64	Aerobic vs control	3 sessions/ week, 1h per session	"50-70% intensity"	1 hour	6 weeks

Tomar et al[33]	Isokinetics and Exercise Science	2014	22	Aerobic vs control	3x/week	Start at 40-50% maximum heart rate, progress to 60-70% maximum heart rate	60-70 minutes	12 weeks
Tunar et al[65]	Journal of Diabetes and Its Complications	2012	31	Pilates vs control	3x/week	3 sets of 8 pilates exercises, each with 6-10 repetitions	40 minutes	12 weeks
Wong et al[35]	Journal of Clinical Nursing	2011	23	Aerobic vs control	≥3x/week	40-60% heart rate reserve	Start at 10-20 minutes, progress to 20-30 minutes	3 months
