# Supplementary material for: Filling gaps in type 1 diabetes and exercise research: a scoping review and priority-setting project
Source: BMJ Open Diabetes Res Care. 2020 Mar 4;8(1):e001023. doi: 10.1136/bmjdrc-2019-001023 (PMC7059416; doi:10.1136/bmjdrc-2019-001023)
Supplement: Supplementary data [file bmjdrc-2019-001023supp003.pdf]

SUPPLEMENTARY 3: OUTCOMES ASSESSED IN RANDOMIZED TRIALS OF EXERCISE FOR PATIENTS WITH TYPE 1 DIABETES

	THEME: GLUCOSE CONTROL		THEME: INSULIN REQUIREMENTS	THEME: ANTHROPOMETRICS			THEME: FITNESS AND EXERCISE CAPACITY					THEME: BLOOD MARKERS OF HEALTH				THEME: COMPLICATIONS				THEME: SUBJECTIVE MEASURES			
	HbA1c	Fasting Glucose	Insulin Dose	Body Weight / BMI	Waist Circumference	Body Composition	VO2 Max / Peak	Energy Expenditure	Daily Steps	Sedentary/ Physical Activity Time	Other Fitness	Blood Lipids	Apolipoproteins	Leptin	Markers of Bone Turnover	Hypoglycemia	Diabetic Neuropathy	Left Ventricular Heart Volume	Endothelial Function	Quality of Life	Mental Health	Physical Activity Intentions	Physical Activity Efficacy
Aouadi et al 2011	✓		✓	✓								✓				✓							
Balducci et al 2006																	✓						
Brazeau et al 2014	✓			✓			✓	✓	✓	✓		✓				✓						✓	✓
Boff et al 2019	✓						✓									✓			✓				
D'hooge et al 2011	✓	✓	✓	✓	✓	✓	✓				✓					✓				✓			
Gusso et al 2017	✓		✓	✓		✓												✓					
Heyman et al 2007				✓		✓					✓	✓	✓	✓						✓			
Laaksonen et al 2000	✓			✓		✓						✓	✓										
Maggio et al 2012				✓		✓									✓								
Mohammed et al 2016		✓		✓													✓						
Quirk et al 2018																✓							
Roberts et al 2002	✓			✓			✓																
Salem et al 2010	✓		✓	✓	✓							✓				✓							
Sigal et al 2011	✓											✓				✓							
Sigal et al 2012	✓				✓						✓	✓				✓							
Talakoub et al 2012																					✓		
Tomar et al 2014	✓		✓									✓				✓							
Tunar et al 2012	✓		✓	✓							✓	✓											
Wong et al 2011	✓						✓													/			
**Total # of Studies Reporting**	**13**	**2**	**6**	**11**	**3**	**5**	**5**	**1**	**1**	**1**	**4**	**9**	**2**	**1**	**1**	**9**	**2**	**1**	**1**	**2**	**1**	**1**	**1**
Reporting Significant Positive Effect	2	0	4	3	2	3	1	0	0	0	4	5	2	0	0	0	2	1	1	1	1	0	0
Reporting No Signficiant Effect	11	2	2	6	1	2	4	1	1	1	0	4	0	1	0	6	0	0	0	0	0	1	1
Reporting Significant Negative Effect	0	0	0	2	0	0	0	0	0	0	0	0	0	0	1	3	0	0	0	0	0	0	0

NOTE: "✓" indicates variable was measured and reported in the study; "/" indicates variable was measured but not reported

1
