# Supplementary material for: Filling gaps in type 1 diabetes and exercise research: a scoping review and priority-setting project
Source: BMJ Open Diabetes Res Care. 2020 Mar 4;8(1):e001023. doi: 10.1136/bmjdrc-2019-001023 (PMC7059416; doi:10.1136/bmjdrc-2019-001023)
Supplement: Supplementary data [file bmjdrc-2019-001023supp004.pdf]

SUPPLEMENTARY 4: STUDY DETAILS OF RANDOMIZED TRIALS OF EXERCISE WITH PATIENTS WITH TYPE 1 DIABETES REGISTERED WITHIN A REGISTRY

**Registration Information**				**Intervention Details**				**Measured Outcomes**	
Principal Investigator	Registration #	Status	Last Update	Sample Size	Type	Duration	Frequency	Primary	Secondary
Pierre Fontaine	clinicaltrials.gov: NCT03528226	Not yet recruiting	15/Oct/18	34	Aerobic	4 months	2x supervised, 1x un-supervised per week	1. Flow mediated dilation	1. Vascular responses<br>2. $VO2_{max}$<br>3. Blood nitric oxide and neurotrophic factors<br>4. Body composition
Dominique Daurman	clinicaltrials.gov: NCT03199638	Unknown	27/Jun/17	24	Exercise snacks	3 months	21x/week (3x/day)	1. HbA1c<br>2. MAGE<br>3. % time blood glucose in/above/ below target range<br>4. Insulin sensitivity<br>5. Insulin dose	N/A

Ramon Baron	WHO Registry: ISRCTN12066515	Complete	1/Jul/19	29	Resistance	32 weeks	2x/week	1. HbA1c<br>2. Physical fitness (6 minute walk test and strength tests)	1. Adiponectin levels<br>2. Intima media thickness of left carotid artery
Palak Kishorbhai Purohit	WHO Registry: CTRI/2018/03/012 270	Not yet recruiting	5/Mar/18	100	Yoga	12 weeks	Not specified	1. Signs/ symptom s of juvenile diabetes	1. Prevention of complications<br>2. General health status<br>3. Insulin dose<br>4. Quality of life
